# Deep learning algorithm-based multimodal MRI radiomics and pathomics data improve prediction of bone metastases in primary prostate cancer

**DOI:** 10.1007/s00432-023-05574-5

**Published:** 2024-02-05

**Authors:** Yun-Feng Zhang, Chuan Zhou, Sheng Guo, Chao Wang, Jin Yang, Zhi-Jun Yang, Rong Wang, Xu Zhang, Feng-Hai Zhou

**Affiliations:** 1 The First Clinical Medical College of Gansu University of Chinese Medicine, Lanzhou, 730000 China; 2 The First Clinical Medical College of Lanzhou University, Lanzhou, 730000 China; 3 Department of Nuclear Medicine, Gansu Provincial Hospital, Lanzhou, 730000 China; 4 Department of Urology, Gansu Provincial Hospital, Lanzhou, 730000 China

**Keywords:** Prostate cancer, Bone metastasis, Radiomics, Pathomics, Machine learning, Deep learning

## Abstract

**Purpose:**

Bone metastasis is a significant contributor to morbidity and mortality in advanced prostate cancer, and early diagnosis is challenging due to its insidious onset. The use of machine learning to obtain prognostic information from pathological images has been highlighted. However, there is a limited understanding of the potential of early prediction of bone metastasis through the feature combination method from various sources. This study presents a method of integrating multimodal data to enhance the feasibility of early diagnosis of bone metastasis in prostate cancer.

**Methods and materials:**

Overall, 211 patients diagnosed with prostate cancer (PCa) at Gansu Provincial Hospital between January 2017 and February 2023 were included in this study. The patients were randomized (8:2) into a training group (*n* = 169) and a validation group (*n* = 42). The region of interest (ROI) were segmented from the three magnetic resonance imaging (MRI) sequences (T2WI, DWI, and ADC), and pathological features were extracted from tissue sections (hematoxylin and eosin [H&E] staining, 10 × 20). A deep learning (DL) model using ResNet 50 was employed to extract deep transfer learning (DTL) features. The least absolute shrinkage and selection operator (LASSO) regression method was utilized for feature selection, feature construction, and reducing feature dimensions. Different machine learning classifiers were used to build predictive models. The performance of the models was evaluated using receiver operating characteristic curves. The net clinical benefit was assessed using decision curve analysis (DCA). The goodness of fit was evaluated using calibration curves. A joint model nomogram was eventually developed by combining clinically independent risk factors.

**Results:**

The best prediction models based on DTL and pathomics features showed area under the curve (AUC) values of 0.89 (95% confidence interval [CI], 0.799–0.989) and 0.85 (95% CI, 0.714–0.989), respectively. The AUC for the best prediction model based on radiomics features and combining radiomics features, DTL features, and pathomics features were 0.86 (95% CI, 0.735–0.979) and 0.93 (95% CI, 0.854–1.000), respectively. Based on DCA and calibration curves, the model demonstrated good net clinical benefit and fit.

**Conclusion:**

Multimodal radiomics and pathomics serve as valuable predictors of the risk of bone metastases in patients with primary PCa.

**Supplementary Information:**

The online version contains supplementary material available at 10.1007/s00432-023-05574-5.

## Introduction

According to 2022 American Cancer Society statistics, Prostate cancer (PCa) is the top-ranking cancer and second leading cause of death among men (Siegel et al. 2022). Bone metastasis is a frequently observed consequence of the progressive development of prostate cancer. Patients with bone metastasis in PCa tend to experience a significantly worse prognosis (Chen et al. 2016). Studies have indicated that patients with PCa who develop bone metastases have significantly higher mortality rates than those without bone metastases. The median survival rates and tumor-specific survival rates with bone metastases reduced to 24 and 32 months, respectively, and the chance of mortality was shown to be 1.5 times higher in patients with bone metastases compared to those with lymph node metastases (Gandaglia et al. 2015). Moreover, early bone metastases in most patients with PCa lack typical clinical manifestations; Consequently, the absence of efficient early diagnostic methods frequently results in a delay in the initiation of treatment.

A whole-body bone scan is currently the primary imaging method for detecting bone metastases early in PCa. However, the standard criteria for using a whole-body bone scan to screen for bone metastases are lacking. In clinical practice, the presence of bone metastases foci is predicted based on symptoms and clinical indicators, such as PSA levels, GS, body mass index, and AAPR (ALB/ALP). However, the sensitivity and specificity of the indicators are poor (Gillessen et al. 2018; Janssen et al. 2020; Karademir et al. 2013; Chang et al. 2014). Therefore, it is necessary to explore and identify simpler and more effective indicators to predict the risk of bone metastases in PCa. This will help guide treatment in clinical practice.

Artificial intelligence (AI) technologies are being widely adopted in medical research and have shown significant benefits in various aspects of cancer management, including preoperative diagnosis, prognostic evaluations, and prediction of survival. In recent years, several studies have demonstrated success in predicting bone metastases in PCa through clinical indicators and imaging techniques (Liao et al. 2018; Hannan et al. 2019). Zhang et al. (2020) demonstrated the effectiveness of radiomics in predicting bone metastases in patients with PCa, with significantly improved prediction accuracy when combined with clinical indicators. This offers valuable insights to clinicians in cancer treatment and management.

Radiomics is centered around the proficient extraction of quantitative image features to precisely depict the areas affected by lesions effectively. These radiomics features represent tissue and lesion characteristics and can be integrated with laboratory results, histopathology, genomics, and other histological data via machine learning algorithms. They play a crucial role in solving a range of clinical issues, including precise disease diagnosis, treatment evaluation, and prognosis prediction (Lambin et al. 2017; Aerts et al. 2014; Gillies et al. 2016). With advancements in AI, deep learning (DL) is making its way into medical research. DL is an advanced machine learning algorithm that is a subfield of AI. It mimics the neural connections in the human brain, enabling the learning and extraction of complex high-level features from input data through multilayer neural networks. This ability renders automated classification, recognition, and prediction of data feasible (Tran et al. 2021).

Pathomics is an innovative approach that combines pathology, imaging, and computer science to understand disease processes. The use of pathomics has revolutionized the diagnosis and treatment of diseases by digitizing and automating the analysis of histological images using computer vision and machine learning algorithms. Through digital images, computers can identify and extract entities, such as cells and blood vessels, and classify and characterize them. This information improves disease classification, grading, prognostic evaluations, and treatment planning. Pathomics also has the potential to reveal the structure and arrangement of tumor cells and the tissue microenvironment (Chen et al. 2022). It has been shown to be effective in predicting the diagnosis and prognosis of various cancers, including bladder, gastric, and liver (Chen et al. 2021, 2023; Hindson 2023; Qu et al. 2023).

To help with the prediction of bone metastasis in primary PCa, we developed a model based on radiomics and pathomics data and explored its clinical application in PCa.

## Materials and methods

This retrospective study received approval from the Ethics Committee of Gansu Provincial Hospital (Approval ID: 2021-260) and was exempted from obtaining informed consent. Moreover, the research project strictly adhered to the AI model training specifications provided by the Lanzhou University unit.

### Participants

**Fig. 1:**
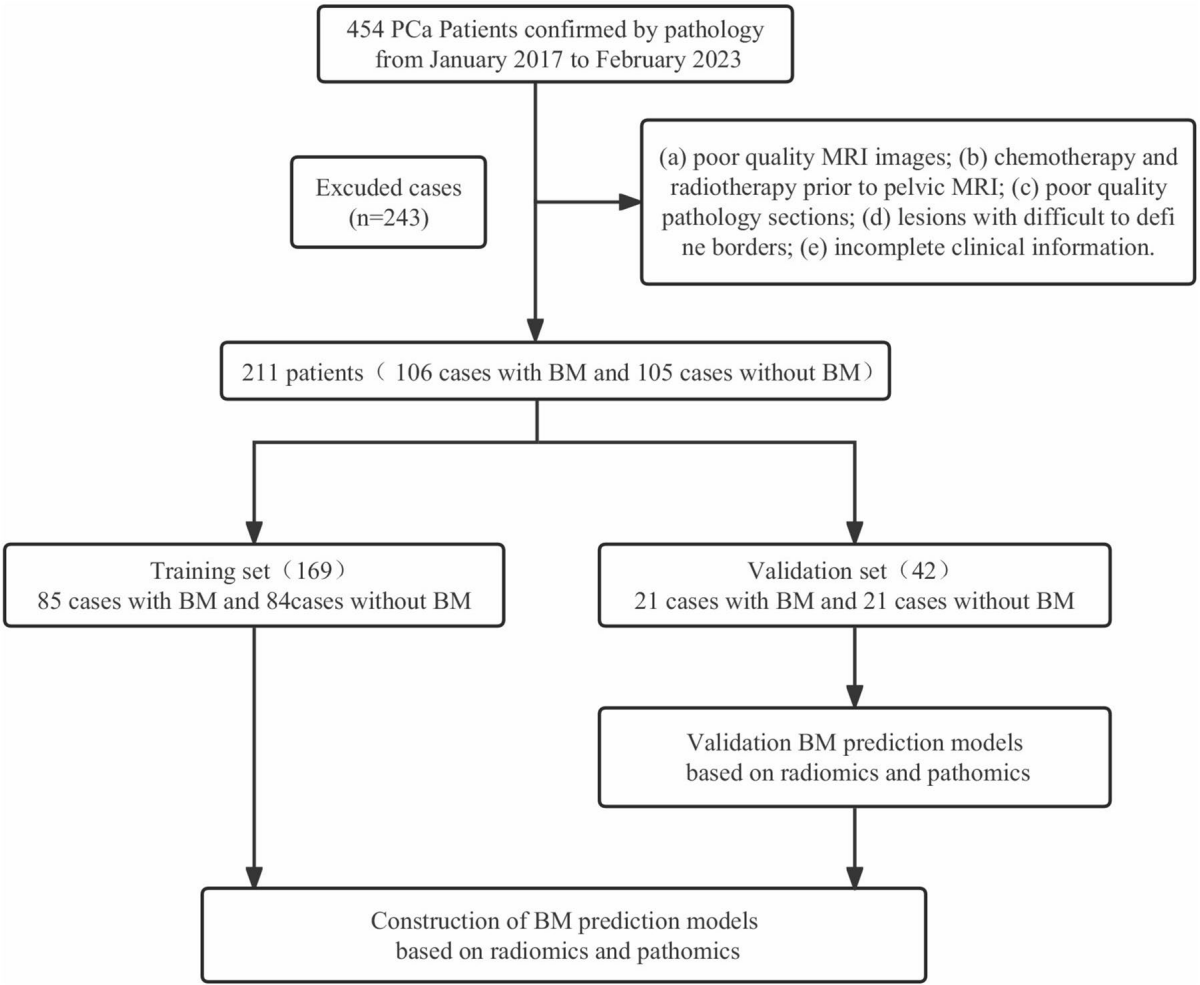
The flow chart for the exclusion of patients. *BM, bone metastases

Our retrospective cohort study screened 454 patients diagnosed with PCa between January 2017 and February 2023 at Gansu Provincial Hospital. The inclusion criteria were (a) patients with a first accepted pathological diagnosis of PCa, (b) magnetic resonance imaging (MRI) scans conducted within 30 days of PCa diagnosis to avoid confounding effects of medications on measurements, (c) absence of primary pelvic bone diseases, such as primary osteosarcoma, bone cysts, hematological disease, and fractures, (d) availability of complete prognostic information, (e) no missing information on whole-body bone visualization, and (f) availability of pathology tissue sections. The exclusion criteria were (a) poor-quality MRI images that hindered the identification of the exact tumor location, (b) patients who had received chemotherapy or radiotherapy prior to pelvic MRI, (c) poor-quality pathology sections with non-uniform staining, (d) lesions with unclear boundaries, and (e) incomplete clinical information. Figure 1 illustrates the patient recruitment process.

### Prostate tumor segmentation

The regions of interest (ROIs) were segmented from T2WI, DWI, and ADC modalities by an experienced radiologist (R.W) and a seasoned urologist (FH.Z) who specialized in interpreting pelvic and prostate MRI, respectively. The physicians were blinded to the presence of bone metastases before labeling. The ITK-SNAP software was used for the labeling process. In cases of disagreement regarding specific tumor lesions, a consensus was attained after a discussion between the two experts. The original image files and ROI files were saved for the extraction of radiologic features. In addition, three-dimensional (3D) ROIs were cropped for the extraction of deep transfer learning (DTL) features. To ensure data quality, N4 bias correction was performed on all images before segmentation. The Digital Imaging and Communications in Medicine (DICOM) standard file format, which is commonly used for managing medical imaging information and related data, was normalized to a resampled format with a resolution of 1 mm × 1 mm × 1 mm.

One pathologist (Z.X) retrospectively collected hematoxylin and eosin (H&E)-stained pathology sections of patients with primary PCa. From this collection, typical PCa pathology sections measuring 20 × 10 times were selected. The sections were then divided into multiple pieces using a cropping tool to remove the white background. To ensure data consistency, these patches in jpg format underwent pixel normalization and were resampled to a standardized resolution of 448 × 448. This standardization was done to facilitate the extraction of pathomics features.

### Feature extraction

After marking the ROIs, radiomics features were extracted using PyRadiomics (http://www.radiomics.io/pyradiomics.html), and non-task layer avgpool features were extracted using the ResNet 50 model. A total of 2553 radiomics features and 6144 DTL features were extracted. To obtain a final set of 3379 features, the features with field 0 were removed from the DTL set. In addition, 2048 pathomics features were extracted from each patch, and these features were averaged across all patches to derive the pathomics features for each patient.

### Feature selection and signature construction

Z scores were utilized to standardize the dataset $\left( {\frac{{{\text{column}} - {\text{mean}}}}{{{\text{std}}}}} \right)$, while Spearman’s correlation coefficient was used to assess inter-observer agreement for feature extraction. Features with a correlation coefficient above 0.9 were deemed reliable and were used to create a feature set for subsequent analysis. The least absolute shrinkage and selection operator (LASSO) algorithm was then applied in a stepwise search to identify the best combination of accuracy-based features. Multiple iterations were performed to evaluate the importance of each feature. For determining hyperparameters, such as the number of features, a fivefold cross-validation method was applied to the training dataset. Various classifiers, including LR, SVM, NaiveBayes, XGBoost, MLP, KNN, ExtraTrees, LightGBM, and GradientBoosting, were utilized to construct predictive models for radiomics and pathomics.

### Model evaluation

To evaluate the predictive performance of the model, we plotted receiver operating characteristic (ROC) curves and calculated their corresponding area under the curve (AUC) values. Furthermore, we employed decision curve analysis (DCA) curves and calibration curves to assess the net clinical benefit and goodness of fit of the model. Finally, we generated a nomogram incorporating clinical indicators, radiomics features, DTL features, and pathomics features.

### Statistical analysis

Statistical analyses were conducted using the Statistical Package for Social Sciences (SPSS) 23.0 and R statistical software. The Kolmogorov–Smirnov test was used to assess the normality of the measures. Measures that followed a normal distribution were reported as Mean ± standard deviation, (*x* ± *s*), whereas those that did not conform to a normal distribution were reported as the median (upper and lower quartiles). For comparing the measures, an independent samples *t* test was used when the data were normally distributed and had equal variance. The Mann–Whitney *U* test was utilized in cases of skewed distribution or unequal variance of data. To construct the prediction model and create the nomogram, a multi-factor logistic regression analysis was conducted to identify independent predictors. The discriminative power of the model was evaluated using the AUC of the ROC. In addition, the clinical value of the model was assessed by plotting DCA. A *p* value of < 0.05 was considered statistically significant to detect meaningful differences.

## Results

### Clinical characteristics

**Fig. 2:**
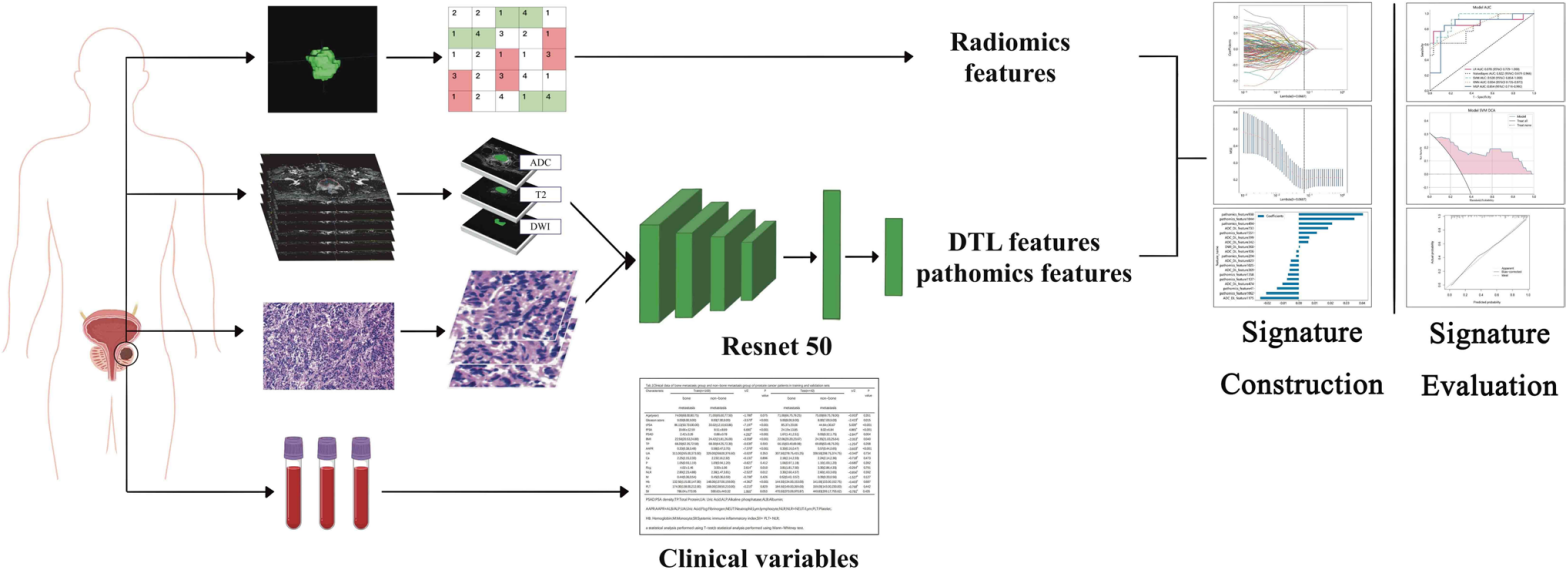
The schematic outline of the study

The study flow is depicted in Fig. 2. A total of 243 participants met the exclusion criteria, and 211 patients were included in the study. Among the included patients, 106 were classified into the bone metastasis group based on the results of a whole-body bone scan, while the remaining 105 patients were categorized into the non-bone metastasis group.

The basic clinical characteristics of the patients in the training and validation groups are presented in Table 1. Statistical analysis revealed no significant difference in the incidence of bone metastases between the two groups.

**Table 1:** Comparison of clinical data of prostate cancer patients in training set and validation set

Characteristic	Train (*n* = 169)	Test (*n* = 42)	*t*/*Z*	*p* value
Age (year)	73.00 (66.00.78.00)	74.00 (67.00.78.25)	− 0.438^b^	0.661
Gleason score	8.00 (8.00.9.00)	8.00 (8.00.9.00)	− 0.522^b^	0.602
tPSA	58.36 (27.07, 100.00)	75.65 (36.35, 100.00)	− 1.293^b^	0.196
fPSA	9.06 (3.23.28.51)	12.74 (3.27, 30.00)	− 0.829^b^	0.407
PSAD	1.09 (0.49.1.92)	1.49 (0.76, 2.28)	− 1.315^b^	0.189
BMI	23.53 (21.12.25.35)	23.14 (20.50, 25.23)	− 1.045^b^	0.296
TP	68.30 (63.35, 72.50)	67.00 (63.43, 72.75)	− 0.048^b^	0.962
AAPR	0.47 (0.31.0.61)	0.44 (0.31.0.58)	− 0.661^b^	0.509
UA	320.00 (265.00, 374.50)	334.50 (288.75, 383.25)	− 0.623^b^	0.533
Ca	2.25 (2.16.2.32)	2.23 (2.14.2.35)	− 0.205^b^	0.838
P	1.07 (0.93.1.19)	1.08 (0.98.1.19)	− 0.298^b^	0.766
Fbg	3.46 (2.84, 4.35)	3.30 (2.83, 4.38)	− 0.315^b^	0.753
NLR	2.62 (1.80.4.19)	2.79 (1.65, 4.07)	− 0.088^b^	0.930
M	0.44 (0.36, 0.56)	0.44 (0.35, 0.57)	− 0.023^b^	0.982
Hb	137.49 + 25.22	142.86 ± 16.33	− 1.687^a^	0.095
PLT	173.00 (139.00.210.00)	175.00 (143.00, 233.75)	− 0.851^b^	0.395
SII	508.65 (271.97, 773.12)	463.56 (346.01.927.75)	− 0.572^b^	0.567

*PSAD* PSA density; *TP* Total Protein; *UA* Uric Acid; *ALP* Alkaline phosphatase; *ALB* Albumin; *AAPR* AAPR = ALB/ALP; *UA* Uric Acid; *Fbg* Fibrinogen; *NEUT* Neutrophil; *Lym* lymphocyte; *NLR* NLR = NEUT/Lym; *PLT* Platelet; *HB* Hemoglobin; *M* Monocyte; *SII* Systemic immune inflammatory index, SII = PLT* NLR;

^a^statistical analysis performed using *t* test

^b^statistical analysis performed using Mann–Whitney *U* test

### Feature selection and signature construction

**Fig. 3:**
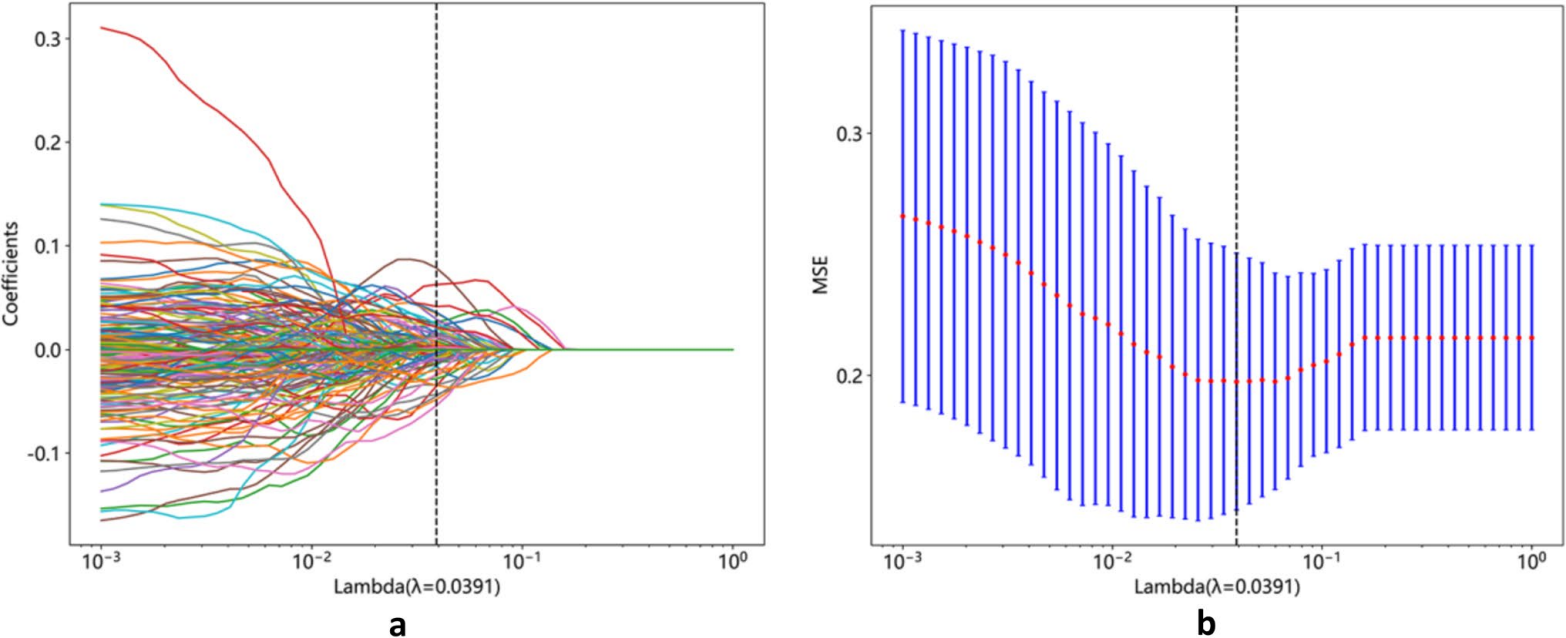
**a** The LASSO coefficient profiles of the features. Different color line shows corresponding coefficient of each feature; **b **the tuning parameter (*λ*) selection in LASSO model

**Fig. 4:**
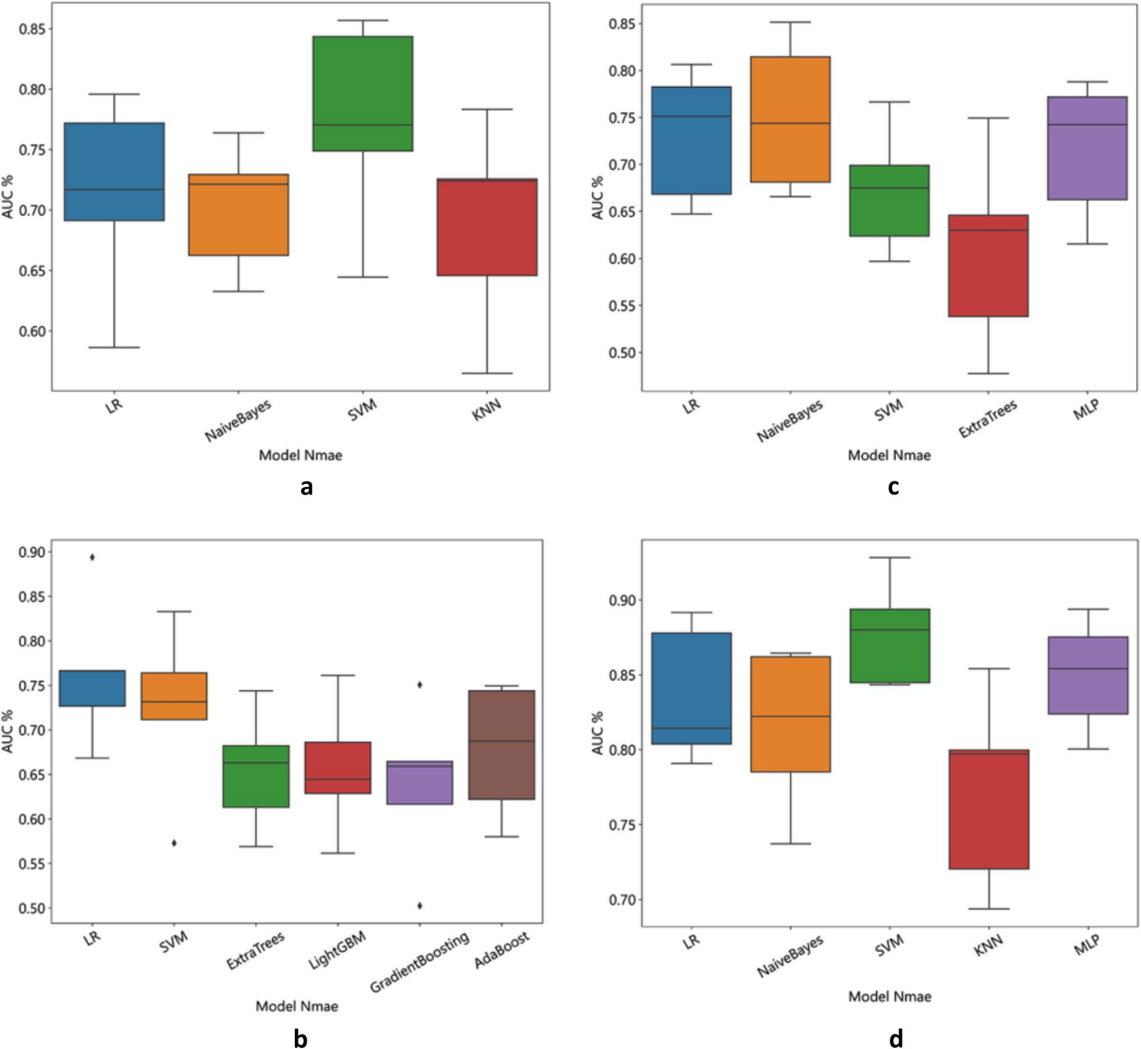
The accuracy based on various classifiers: **a **radiomics features; **b** DTL features; **c **pathomics features; **d **combined model

A total of 2553 radiomics features and 3379 DL features were extracted from the T2WI, DWI, and ADC images of each patient, and 2048 pathomics features were extracted from each H&E-stained sections. To determine the hyperparameters, including the number of features in the model, we performed fivefold cross-validation on the training dataset (Fig. 3a). Utilizing the LASSO regression model (Fig. 3b), we identified 44 radiomics features, 23 DTL features, and 13 pathomics features with non-zero coefficients, which were found to be closely associated with bone metastases. Based on the selected features, we constructed prediction models by employing various classifiers (Fig. 4a–d).

### Validation of radiomics and pathomics signature

**Fig. 5:**
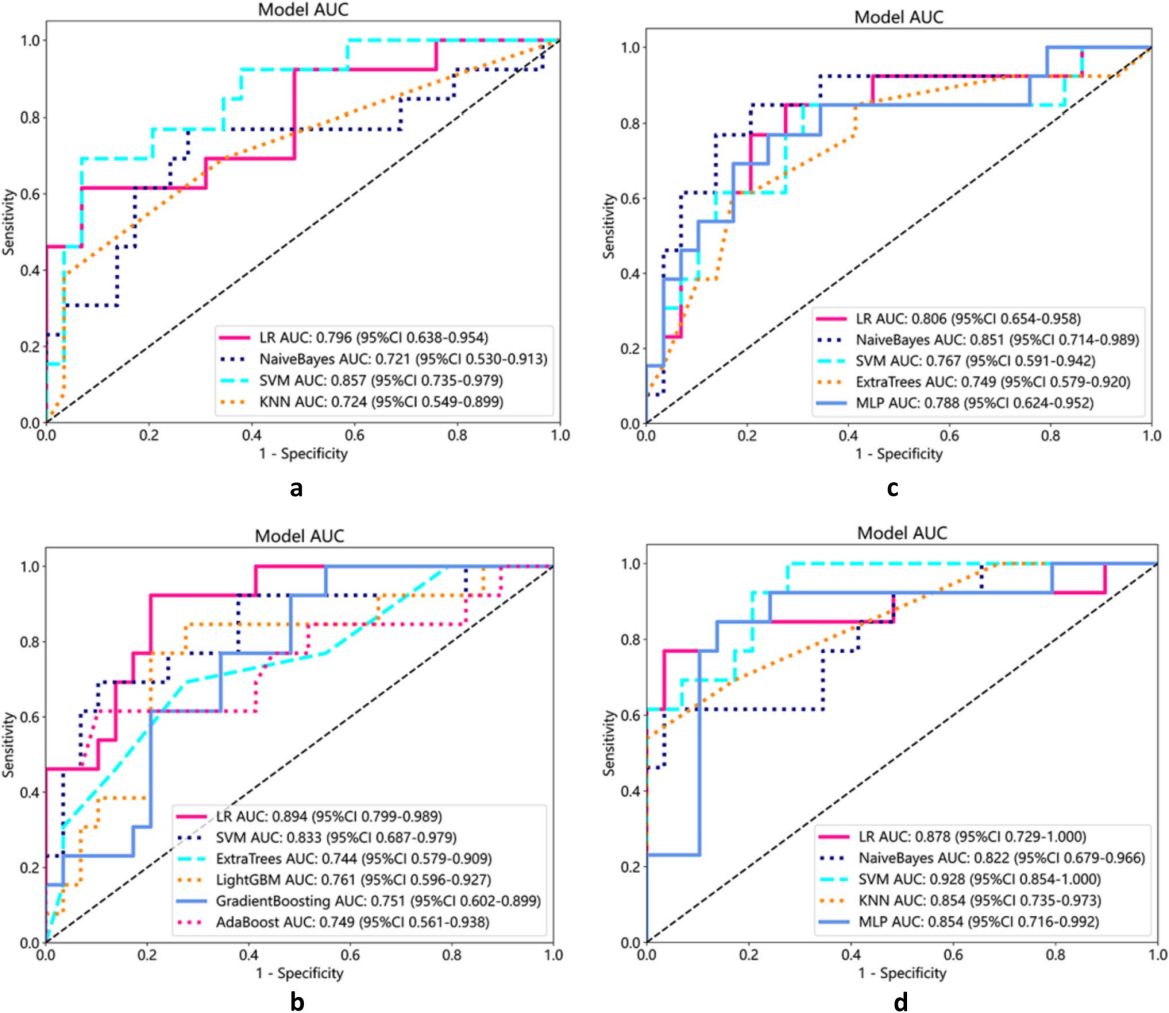
The ROC curve of the prediction model in the validation set. **a** Radiomics features; **b** DTL features; **c **pathomics features; **d **combined model. *ROC, receiver operating characteristic curve; AUC, area under the curve

**Fig. 6:**
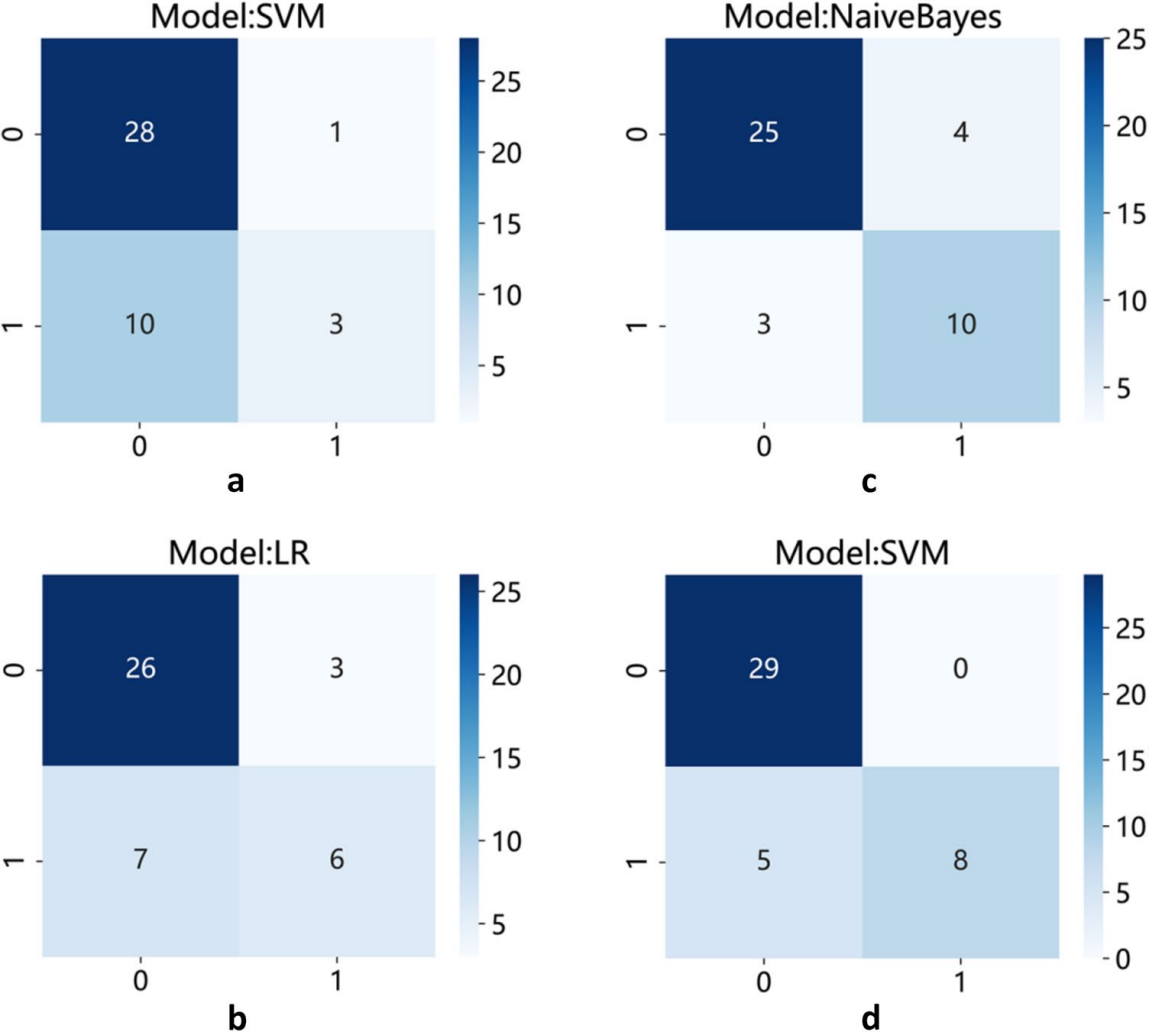
The confusion matrices for the different models. **a** Radiomics features; **b** DTL features; **c** pathomics features; **d** combined model

**Fig. 7:**
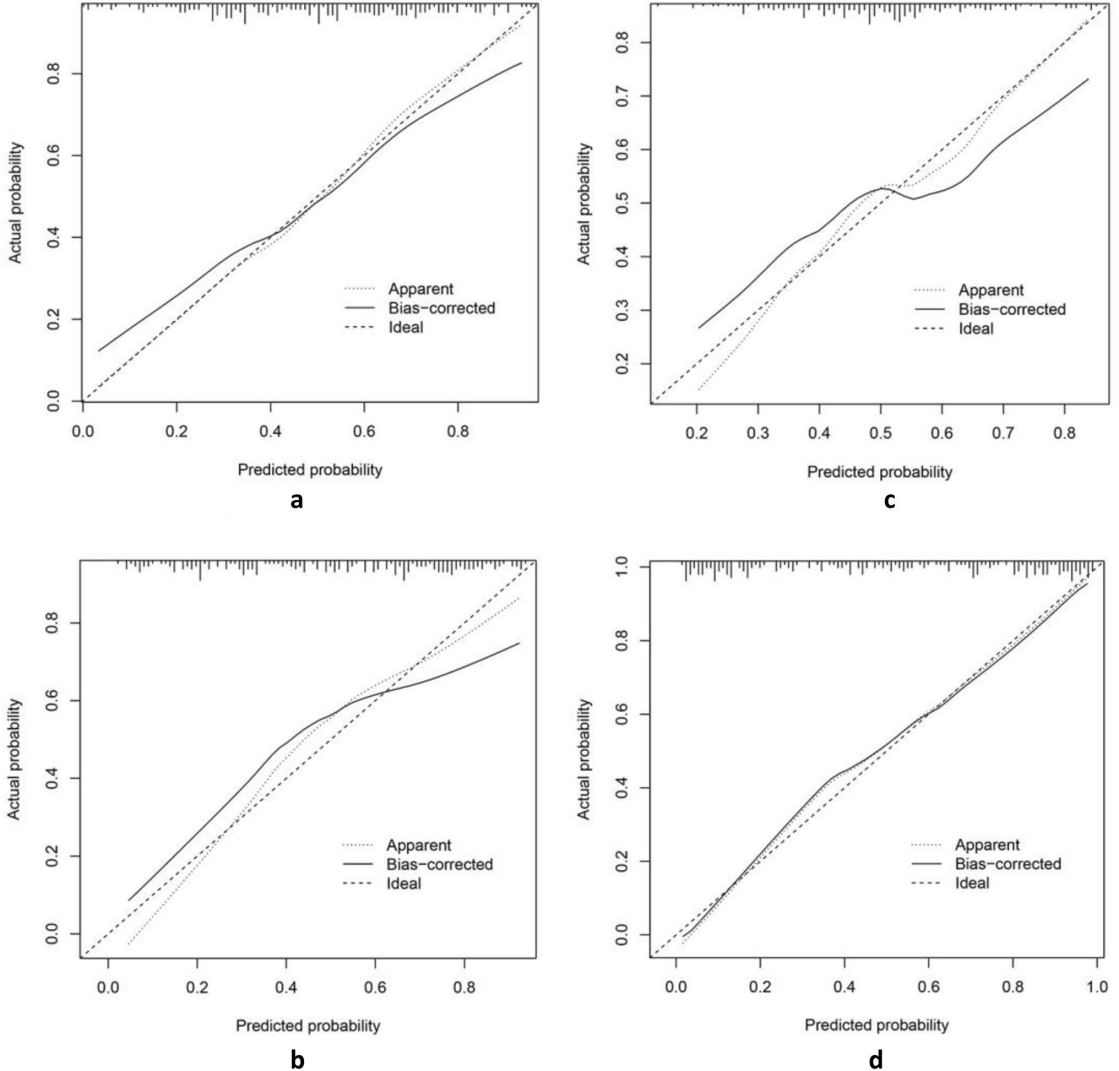
The calibration curve analysis indicates that all models are well-calibrated, with the joint model exhibiting the highest level of calibration. **a** Radiomics features; **b** DTL features; **c** pathomics features; and **d** combined model

The best predictive model for extracting radiomics features using PyRadiomics for bone metastasis in PCa was found to be the Support Vector Machine (SVM) model, with an AUC value of 0.86 (95% confidence interval [CI], 0.735–0.979; Fig. 5a). Furthermore, the Logistic Regression (LR) model utilizing DTL features demonstrated the best predictive performance, with an AUC value of 0.89 (95% CI, 0.799–0.989; Fig. 5b). The Naive Bayes model showed the highest predictive capability for pathomics features, with an AUC value of 0.85 (95% CI, 0.714–0.989, Fig. 5c). Finally, the most effective predictive model, combining radiomics features, DTL features, and pathomics features using the SVM model, provided an AUC value of 0.93 (95% CI, 0.854–1.000; Fig. 5d). The confusion matrices for the different models are presented in Fig. 6a–d, and the calibration curves are shown in Fig. 7a–d. This enabled the assessment of the calibration performance of the models.

### Nomogram construction and validation

**Fig. 8:**
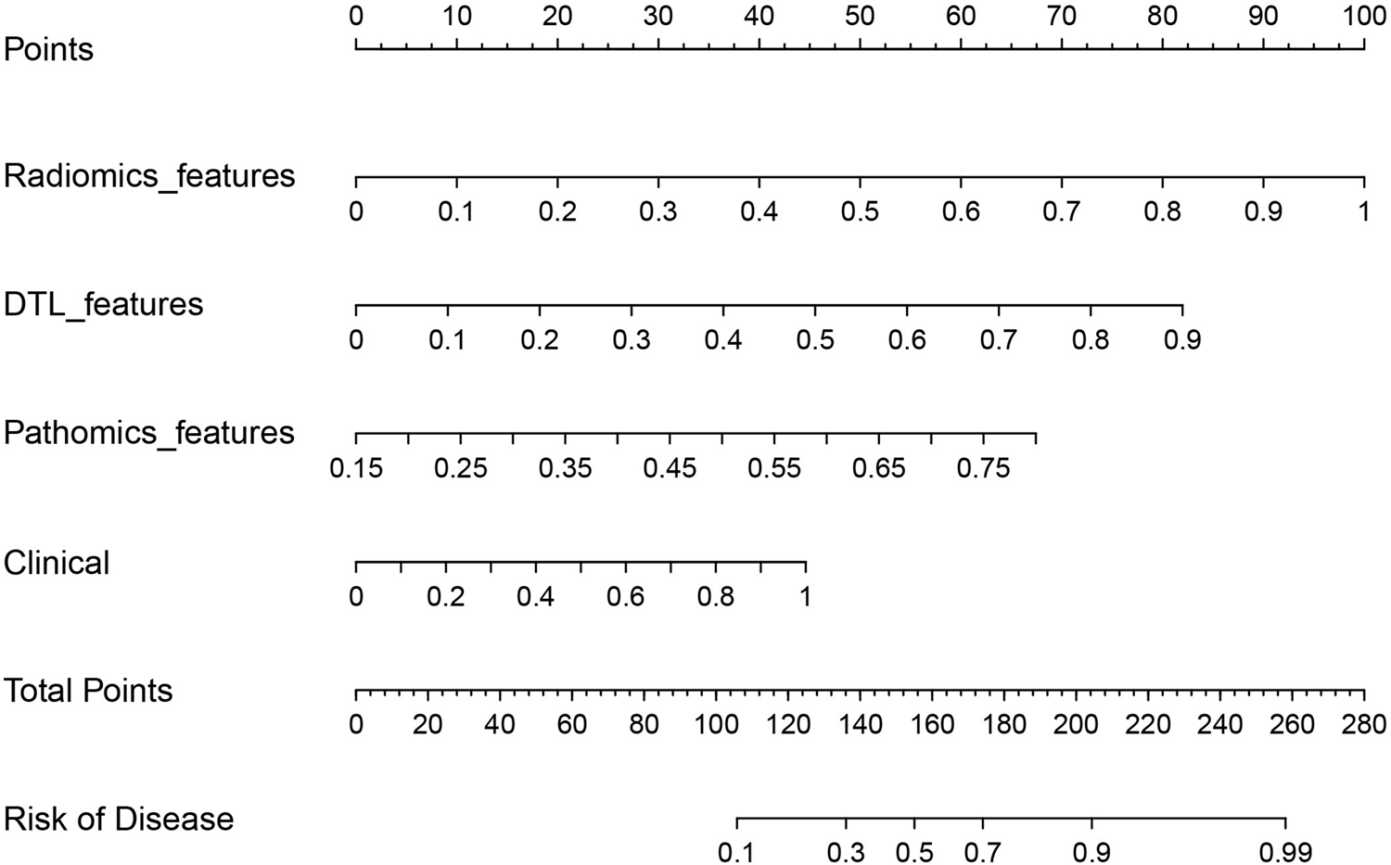
The nomograms based on different model features

The nomogram demonstrated the enhanced diagnostic performance of radiomics and pathomics models compared to the clinical models (Fig. 8). Therefore, the integration of multi-omics models provides an improved predictive ability for determining the status of bone metastases in PCa.

### Clinical use

**Fig. 9:**
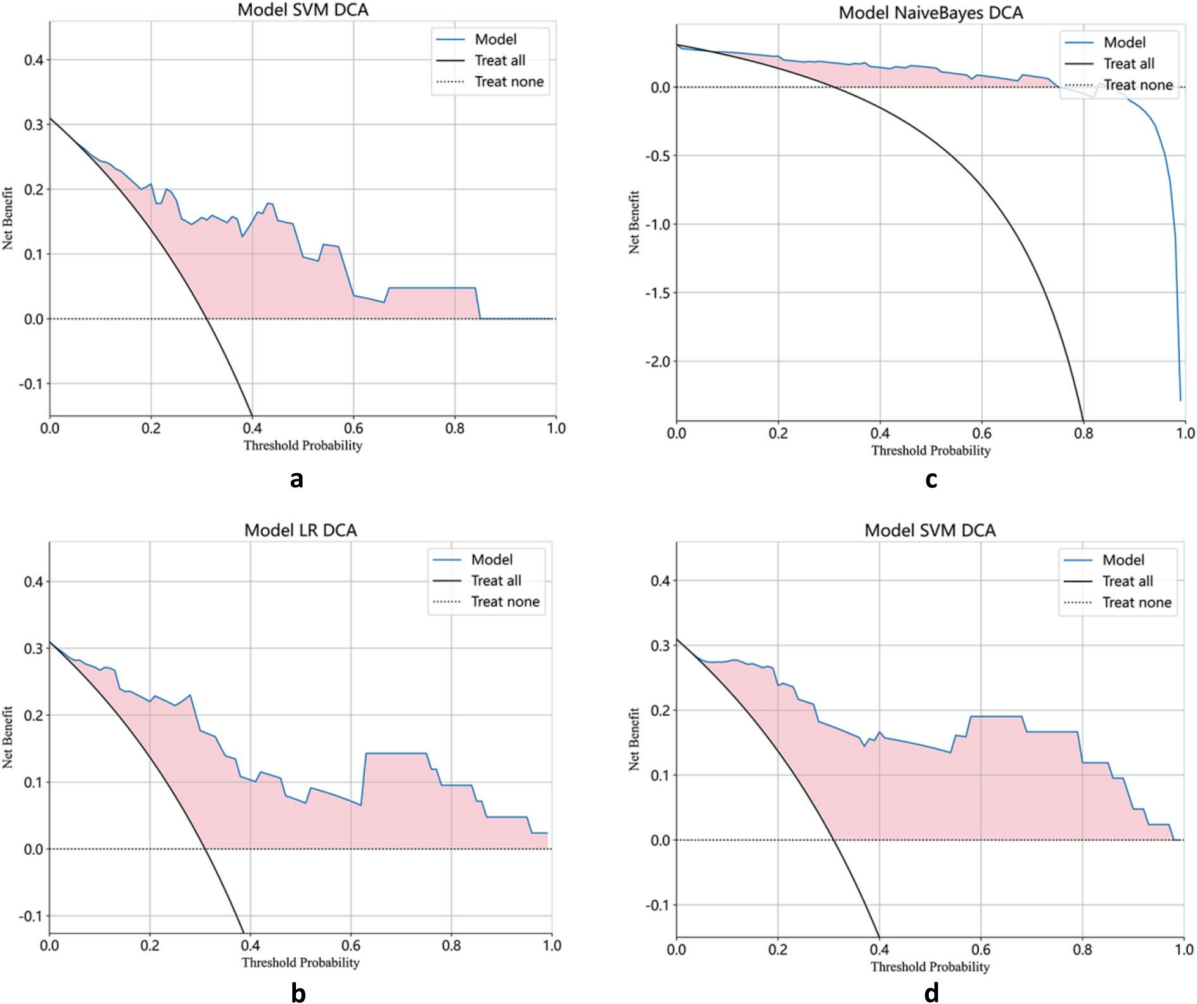
The decision curves (DCA) show that the models all have a good net clinical benefit. **a** Radiomics features; **b** DTL features; **c** pathomics features; and **d** combined model

**Fig. 10:**
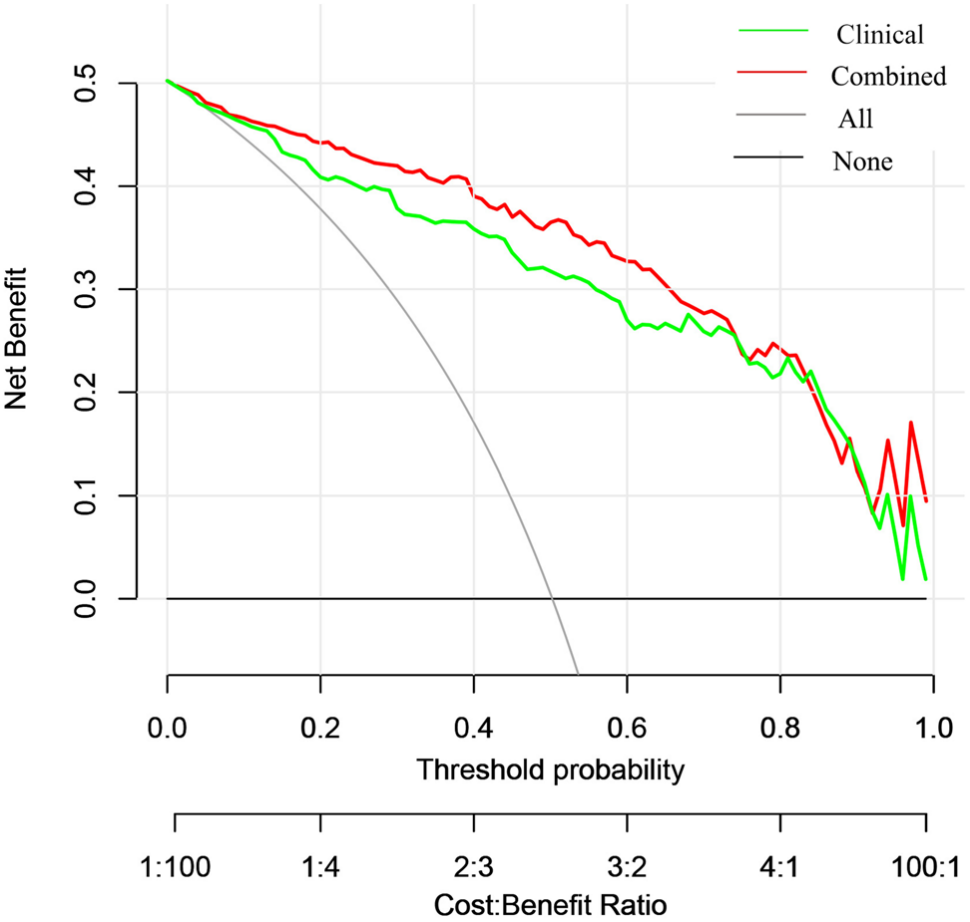
The DCA curve analysis, based on the nomogram model, demonstrated that the combined model yielded superior clinical benefits

The DCA curves indicate a favorable net clinical benefit of both the radiomics and pathomics prediction models using the DTL features (Fig. 9a–d). Also, the DCA curves for the nomograms showed a superior net clinical benefit for the combined models (Fig. 10), providing valuable guidance for clinicians in formulating treatment strategies.

## Discussion

In this study, a radiomics feature was developed and validated to assess the bone metastasis status of PCa through quantitative analysis of MRI images. In addition, DL features were extracted from both MRI and histopathological images and analyzed to demonstrate their relationship with bone metastasis in PCa, independent of traditional clinical and pathological risk factors. Importantly, data from different sources were integrated, and a combined model was constructed that significantly improved the prediction of bone metastasis in PCa patients.

DL has been extensively employed in PCa research. Bulten et al. (2020) developed an automated DL system that showed efficacy with comparable performance to assist pathologists in GS grading. Likewise, Hiremath et al. (2021) demonstrated that DL algorithms can effectively identify clinically significant PCa through MRI. Wang et al. also constructed prediction models for bone metastases in primary PCa based on radiomics features and clinical risk factors; the AUC values were 0.87 and 0.84, respectively (Zhang et al. 2020; Wang et al. 2019). These results align well with our study, which showed an AUC of 0.86. Furthermore, we constructed prediction models using DTL features and pathomics features, and their best models yielded AUC values of 0.89 and 0.85, respectively. Lastly, we integrated radiomics features, DTL features, and pathomics features to build a composite model, which yielded the best prediction model with an AUC value of 0.93. The model also demonstrated a good net clinical benefit as indicated by the DCA curve. The calibration curve further confirmed a better fit.

In this study, we utilized ResNet50 as the model for extracting deep learning features. ResNet50 excels at efficiently training deep neural networks, avoiding the issues of gradient vanishing and exploding. As a result, it performs exceptionally well in image classification tasks and can handle larger and more complex datasets. Due to its wide-ranging applications and outstanding performance, ResNet50 has become a benchmark model for many computer vision tasks, finding extensive use in areas such as object detection, image segmentation, and image generation. It has shown remarkable results in the evaluation of breast cancer, gastric cancer, spinal metastasis, and other tumors (Mo et al. 2023; Iwaya et al. 2023; Liu et al. 2023). Furthermore, the features we extract are not task specific and are not dependent on a single task. These features typically have lower dimensions and encode crucial information from the input data, making them suitable for subsequent tasks such as feature visualization and data clustering. They provide valuable insights for data analysis.

Remarkable advancements through the application of multi-omics approaches have been noted in the field of oncology. Wang et al. and Wan et al. demonstrated the effectiveness of models that combined radiomics and pathomics features for predicting the prognosis of colorectal cancer after surgery and the pathological response to neoadjuvant radiation therapy in locally advanced rectal cancer (Chang et al. 2014; Wang et al. 2022). These data highlight the significance of integrating multi-omics techniques in the comprehensive assessment of patients with cancer, aiming to maximize the utilization of multifaceted patient information and its evaluation. Multi-omics encompasses diverse technical tools, including genomics, transcriptomics, proteomics, metabolomics, and pathomics, to gain a comprehensive understanding of the interactions between different molecules within an organism at the cellular and tissue levels (Pan et al. 2022; Lu et al. 2021). By harnessing multi-omics data, the disease onset and progression mechanisms can be comprehended more thoroughly, leading to improvements in diagnosis, disease prediction, and the development of personalized treatment plans. For example, Vanguri et al. (2022). have assessed the ability of immunotherapy to predict the response in non-small cell lung cancer by integrating radiology, histopathology, and genomics features. Machine learning methods were utilized to incorporate multimodal features into a predictive model for assessing the risk. The study revealed that the multimodal model achieved an AUC value of 0.80, surpassing the predictive power of any individual variable. Furthermore, Kang et al. (2023). argue that multi-omics offers substantial advantages for conducting a comprehensive evaluation of tumor patients. Integration of imaging phenotypes with multi-omics biological data enables a comprehensive assessment, characterization, and decoding of the tumor microenvironment, facilitating the prediction of patient prognosis. It also enhances the understanding of radiological features, as well as the pathological, physiological, and biological basis of the tumor. Considering the advancements in AI, the clinical applications of multi-omics are expected to broaden further.

There are some limitations in this study. First, the generalizability of the model is limited due to the small sample size and the majority of samples being from a single province. We plan to mitigate this limitation by collecting a larger sample size from multiple centers to provide robust evidence for the clinical application of the model. Second, this study was retrospective and relied on the quality of the collected H&E section images. Due to the unavailability of annotations for pathology slices, we were only able to select the typical tumor area for feature extraction. For a more comprehensive analysis, we intend to collect relevant tumor specimens to obtain Whole Slide Image (WSI).

Nevertheless, our model demonstrates an excellent predictive ability. We are committed to continuously refining and updating our model as the DL algorithm iterates. Our ultimate goal is to provide clinicians with more accurate guidance, ultimately benefiting patients to a great extent.

## Conclusion

In summary, the radiomics and pathomics models developed in this study based on feature extraction using DL algorithms can predict the risk of bone metastases in patients with primary PCa. Having this information may change the clinical management strategy for patients with PCa.

### Supplementary Information

Below is the link to the electronic supplementary material.

Supplementary file1 (PDF 2985 KB)

## Data Availability

All data supporting the findings of this study are available within the paper and its Supplementary material.

## Abbreviations

AUC: Area under the curve
ADC: Apparent diffusion coefficient
ALP: Alkaline phosphatase
ALB: Albumin
AAPR: AAPR = ALB/ALP
BMI: Body mass index
DCA: Decision curve analysis
DICOM: Digital imaging and communications in medicine
DWI: Diffusion weighted imaging
DL: Deep learning
DTL: Deep transfer learning
Extratrees: Extremely randomized trees
EAU: European association of urology
GS: Gleason score
KNN: K-nearest neighbor
MLP: Multilayer perceptron
Lasso: Least absolute shrinkage and selection operator
LightGBM: Light Gradient boosting Machine
LR: Logistic regression
MRI: Magnetic resonance imaging
NaiveBayes: Naive Bayes classifier
ROC: Receiver operating characteristic
PCa: Prostate cancer
PSA: Prostate specific antigen
ROI: Region of interest
SVM: Support vector machine
XGBoost: Extreme gradient boosting
