# Supplementary material for: Deep learning algorithm-based multimodal MRI radiomics and pathomics data improve prediction of bone metastases in primary prostate cancer
Source: J Cancer Res Clin Oncol. 2024 Feb 5;150(2):78. doi: 10.1007/s00432-023-05574-5 (PMC10844393; doi:10.1007/s00432-023-05574-5)
Supplement: Supplementary file 1 — Supplementary file1 (PDF 2985 KB) [file 432_2023_5574_MOESM1_ESM.pdf]

甘肃省人民医院科研伦理意见函

批件编号：2023-355	会议日期：2023 年 08 月 25 日
伦理委员会：甘肃省人民医院医学伦理委员会	
研究方案名称：基于深度学习算法的多模态 MRI 影像组学和病理组学数据改善了初诊前列腺癌骨转移预测	
主要研究者：周逢海	
审查文件：项目具体方法、知情同意书等相关材料	

同意	修改后同意	不同意	终止或暂停	弃权
15 票	0 票	0 票	0 票	0 票

1. 委员会议对研究方案及知情同意书的评审意见：

该项目符合伦理要求。

2. 根据以上意见，委员会对该方案的审查决定如下：

同意√

修改后同意

不同意

终止或暂停试验

3. 该研究进行过程中将接受伦理委员会的持续审查：　是√　　否
4. 审查频度为研究批准之日起每 12 月一次。
5. 伦理委员会有根据实际进展情况改变持续审查频度的权利。

主任委员签名：

医院伦理委员会（盖章）：甘肃省人民医院 医学伦理委员会

日期：2023 年 08 月 25 日

备注：

1.研究过程中，对研究方案和知情同意书等相关文件所作的任何修改，均须得到伦理委员会审查同意后方可实施。

2. 本中心发生的严重不良事件或意外不良事件需向伦理委员会作书面通报，伦理委员会有权根据对其评估做出新的决定。

3. 无论试验开始与否，请在持续审查到期前 1 个月提出再次审查的申请。

4. 试验项目若超过一年，需提交年度跟踪审查报告，当出现可能显著影响试验进行或增加试验风险情况，请申请人及时向伦理委员会提交书面报告。
